# Dapagliflozin improves left ventricular remodeling and aorta sympathetic tone in a pig model of heart failure with preserved ejection fraction

**DOI:** 10.1186/s12933-019-0914-1

**Published:** 2019-08-20

**Authors:** Nannan Zhang, Bin Feng, Xuexing Ma, Kangyun Sun, Guidong Xu, Yafeng Zhou

**Affiliations:** 1 Department of Cardiology, The Affiliated Suzhou Hospital of Nanjing Medical University, 242 Guangji Road, Suzhou, 215008 Jiangsu Province People’s Republic of China; 2 Department of Endocrinology and Metabolism, The First Affiliated Hospital of Soochow University, Suzhou, 215006 Jiangsu Province People’s Republic of China; 3 Department of Cardiology, The First Affiliated Hospital of Soochow University, 188 Shizi Road, Suzhou, 215006 Jiangsu Province People’s Republic of China

**Keywords:** SGLT2 inhibitor, Dapagliflozin, Hypertention, Heart failure with preserved ejection fraction, NO-cGMP-PKG pathway

## Abstract

**Background:**

Heart failure with preserved ejection fraction (HFpEF) is a difficult disease with high morbidity and mortality rates and lacks an effective treatment. Here, we report the therapeutic effect of dapagliflozin, a sodium-glucose cotransporter 2 inhibitor (SGLT2i), on hypertension + hyperlipidemia-induced HFpEF in a pig model.

**Methods:**

HFpEF pigs were established by infusing a combination of deoxycorticosterone acetate (DOCA) and angiotensin II (Ang II), and Western diet (WD) feeding for 18 weeks. In the 9th week, half of the HFpEF pigs were randomly assigned to receive additional dapagliflozin treatment (10 mg/day) by oral gavage daily for the next 9 weeks. Blood pressure, lipid levels, echocardiography and cardiac hemodynamics for cardiac structural and functional changes, as well as epinephrine and norepinephrine concentrations in the plasma and tissues were measured. After sacrifice, cardiac fibrosis, the distribution of tyrosine hydroxylase (TH), inflammatory factors (IL-6 and TNF-α) and NO-cGMP-PKG pathway activity in the cardiovascular system were also determined.

**Results:**

Blood pressure, total cholesterol (TC), triglyceride (TG) and low-density lipoprotein (LDL) were markedly increased in HFpEF pigs, but only blood pressure was significantly decreased after 9 weeks of dapagliflozin treatment. By echocardiographic and hemodynamic assessment, dapagliflozin significantly attenuated heart concentric remodeling in HFpEF pigs, but failed to improve diastolic function and compliance with the left ventricle (LV). In the dapagliflozin treatment group, TH expression and norepinephrine concentration in the aorta were strongly mitigated compared to that in the HFpEF group. Moreover, inflammatory cytokines such as IL-6 and TNF-α in aortic tissue were markedly elevated in HFpEF pigs and inhibited by dapagliflozin. Furthermore, the reduced expression of eNOS and the PKG-1 protein and the cGMP content in the aortas of HFpEF pigs were significantly restored after 9 weeks of dapagliflozin treatment.

**Conclusion:**

9 weeks of dapagliflozin treatment decreases hypertension and reverses LV concentric remodeling in HFpEF pigs partly by restraining sympathetic tone in the aorta, leading to inhibition of the inflammatory response and NO-cGMP-PKG pathway activation.

## Background

With the world population aging, heart failure (HF) has become a very important cause of death in older people [1, 2]. Moreover, HF with preserved ejection fraction (HFpEF) accounts for up to 40–60% of all HF cases, which has become an urgent health problem with high morbidity and mortality [3, 4]. Although considerable studies have been performed to seek effective therapies for HFpEF, it seems that present treatments are not enough to improve the clinical symptoms and prognosis of HFpEF patients [5]. To develop pharmacological treatment strategies for HFpEF, preclinical animal models are essential. Schwarzl and colleagues described a HFpEF pig model induced by deoxycorticosterone acetate (DOCA) and Western diet (WD) feeding, that almost perfectly represents the major features of clinical HFpEF in patients with hypertension, dyslipidemia and physical inactivity [6]. It is not difficlt to see that this model highlights the impact of blood pressure/hyperlipidemia in the pathogenesis (molecular and pathological mechanisms) of HFpEF, and therefore interventions on blood pressure and hyperlipidemia (alone or in combination) might be effective [7]. Additionally, previous studies have suggested that the inflammatory response and a deficient NO-cGMP-PKG signaling pathway in the myocardium are involved in the LV diastolic dysfunction of HFpEF [8–10]. Hence, novel strategies to improve blood pressure, hyperlipidemia, the inflammatory response or the NO-cGMP-PKG signaling pathway may offer a therapeutic benefit to the cardiac function of HFpEF.

Dapagliflozin, a sodium-glucose cotransporter 2 inhibitor (SGLT2i), is a newly developed oral antidiabetic drug that blocks glucose reabsorption via SGLT2 inhibition in the kidney and thus reduces glucose levels in an insulin-independent manner [11]. Some studies have observed that SGLT2 inhibitors reduce systolic blood pressure (SBP) and diastolic blood pressure (DBP) in a broad population of patients with type 2 diabetes mellitus (T2DM) [12, 13]. Moreover, in phase III clinical trials, Zaccardi et al. [14] reported that an SGLT2i increased high-density lipoprotein (HDL) cholesterol levels. Additionally, large, rigorously conducted clinical trials using an SGLT2i, such as empagliflozin for the EMPA-REG OUTCOME trial, canagliflozin for the CANVAS Program and dapagliflozin for the DECLARE trial, found that patients with T2DM at a high risk of cardiovascular events derived cardiovascular benefits from the SGLT2i compared with a placebo [15–17]. Several experimental studies in preclinical rodent models of established T2DM have shown that SGLT2 inhibitors could improve vascular endothelial function [18], decrease oxidative stress, ameliorate myocardial fibrosis and enhance cardiac systolic and diastolic functions [19–21]. However, thus far, it is unclear whether an SGLT2i can improve the cardiac function of HFpEF featured mainly by hypertension, dyslipidemia and physical inactivity, which are the most common causes of HFpEF in the clinic, instead of DM. In fact, there is a growing concern about the role of SGLT2i in the absence of diabetes, which may increase the population that could benefit from SGLT2i [22]. Along this line, we have reasons to speculate that dapagliflozin can be used as a new drug to improve hypertension + hyperlipidemia-induced HFpEF, not only for hypoglycemic action.

Hence in this study, we established a HFpEF pig model with a combination of deoxycorticosterone acetate (DOCA), angiotensin II (Ang II) and Western diet (WD) feeding, that manifests major features of clinical HFpEF patients to a large extent [6, 8, 23], to investigate the therapeutic effects of dapagliflozin on blood pressure, hyperlipidemia, and cardiac structure and function, as well as its potential related molecular mechanisms in HFpEF.

## Materials and methods

### Animals and experimental design

**Fig. 1:**
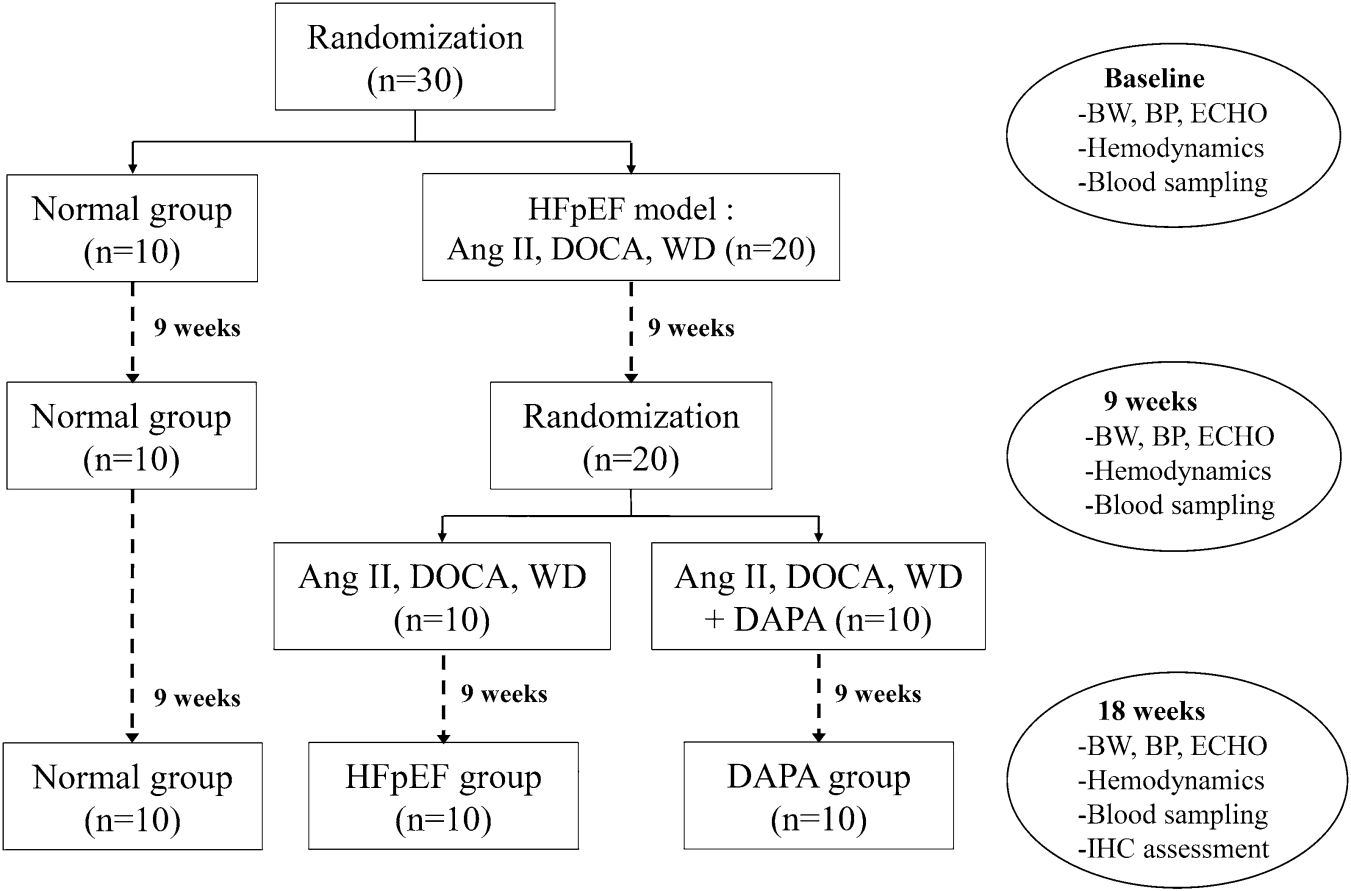
The experimental flow chart of the study. HFpEF: heart failure with preserved ejection fraction; Ang II: angiotensin II; DOCA: deoxycorticosterone acetate; WD: Western diet; DAPA: dapagliflozin; BW: body weight; ECHO: echocardiography; IHC: immunohistochemistry

Thirty-nine-week-old female landrace pigs weighing 30–40 kg were used in this study, ten of which served as normal controls (Normal group), and the remaining twenty pigs were used to establish the HFpEF model. A flow diagram of the experiment is shown in Fig. 1. In brief, twenty pigs underwent implantation of an osmotic infusion pump (Tricumed Medizintechnik GmbH, Delhi, India) to deliver Ang II (0.015 mg/h, Sigma, San Francisco, USA) and were subcutaneously administered DOCA pellets (100 mg/kg, 90-day release depot, Innovative Research of America, Sarasota, USA) at the dorsal aspect of the neck for the first 9 weeks. All HFpEF model pigs were fed a WD (ingredient contents were listed in Additional file 1: Table S1). General anesthesia was induced by tiletamine and zolazepam (Zoletil, 20 mg/kg) and maintained with isoflurane (1–2%), followed by intubation and mechanical ventilation. At the end of the first 9 weeks, all HFpEF pigs were randomized averagely to receive additional dapagliflozin treatment (DAPA group, n = 10) or not (HFpEF group, n = 10) for the next 9 weeks. In the DAPA group, HFpEF pigs were treated with additional dapagliflozin (10 mg/day for 9 weeks, oral gavage; 0.5% hydroxyethylcellulose was used as the vehicle). Dapagliflozin, a selective SGLT2i, was provided by Bristo-Myers Squibb Manufacturing Company (Humacao, Puerto Rico). Blood pressure was measured twice a week, and an echocardiographic assessment was conducted every 2 weeks. Invasive hemodynamic detection was performed at baseline and in the 9th and 18th weeks. At the end of the study, 24 h urine was collected for measurement of volume and glucose, electrolyte, osmolality and uric acid concentrations. All pigs were euthanized and important organs and tissues were immediately dissected and fixed in 10% formaldehyde for histological biochemical determinations or refrigerated at − 80 °C immediately for immunohistochemistry.

### Echocardiographic analysis

A standard transthoracic echocardiogram was performed using a commercially available echocardiographic system (Vivid q, GE Vingmed, Horten, Norway). Standard 2D imaging and M-mode imaging were performed to measure the interventricular septum thickness at end-diastole (IVSd), left ventricular posterior wall thickness (LVPW), LV mass index, and left atrium (LA) diameter and left ventricle (LV) diameters at end-diastole (LVEDD) and systole (LVESD). A pulse-wave Doppler was used to evaluate the isovolumetric relaxation time (IVRT), deceleration time (DT), mitral inflow E wave and mitral inflow A wave. A tissue Doppler was used to measure the median mitral annular early diastolic velocity (e′), followed by the calculation of E/e′ and E/A. The stiffness index β, an index of the elastic property of the arterial wall, was calculated from the blood pressure and the diameter of the aortic root as follows: Stiffness β = Dd/(Ds−Dd) × ln(Ps/Pd), where Ps and Pd were the systolic and diastolic blood pressures, and Ds and Dd were the systolic and diastolic inner diameters of the aortic root, respectively. At least three consecutive beats were taken for each measurement. All analyses were completed off-line in a blind fashion on a workstation (EchoPAC PC, version 6.1.0, GE Vingmed, Horton, Norway).

### Invasive hemodynamic assessment

The invasive hemodynamic study was conducted under general anesthesia at baseline and at the 9th and 18th weeks to asseess cardiac function. The LV ± dp/dt, end-diastolic pressure–volume relationship (EDPVR), end-systolic pressure–volume relationship (ESPVR), left ventricular ejection fraction (LVEF) and cardiac output (CO) were measured with a 7-Fr combined catheter-micromanometer (Millar Instrument, Houston, USA) through the femoral artery. All pressure–volume loop data were recorded at least 8 to 10 beats at end-expiration from the raw LV pressure and conductance volume data using commercially available software (Conduct NT, Leycom, The Netherlands). Right heart catheterization was performed to investigate pulmonary hemodynamic changes using a Swan-Ganz catheter (multilumen, balloon tipped, 110 cm long) conducted under complete aseptic precautions via the right internal jugular vein.

### Biochemical determination

Plasma brain natriuretic peptide (BNP) and Ang II were determined to assess myocardial stretch and renin–angiotensin–aldosterone system (RAAS) system activity, respectively, using available enzyme-linked immunosorbent assay (ELISA) kits (Phoenix Pharmaceuticals, Burlingame, CA, USA). The levels of plasma epinephrine (E) and norepinephrine (NE) were measured to analyze the sympathetic response with commercially available ELISA kits (KA3746, Abnova, Taipei, Taiwan). NE concentrations in the LA, LV, aorta and kidney tissues were also determined (KA3836, Abnova, Taipei, Taiwan). The levels of IL-6, TNF-α and cGMP in the aorta were assessed via available ELISA kits (Abcam, ab-100573, ab-100756 and ab-65356, respectively). Plasma total cholesterol (TC), triglyceride (TG), low-density lipoprotein (LDL), HDL and glycosylated hemoglobin (HbA1c) were measured with specific kits according to the manufacturer’s instructions (Roche Diagnostics, Madison, WI, USA). Urinary electrolyte and osmolality levels were measured by the autoanalyzer CX5 (Beckman Instruments, Fullerton, CA, USA). Urinary glucose and uric acid levels were measured by Cobas Mira autoanalyzer (Roche Diagnostics, Madison, WI, USA).

### Western blot analysis

The aortic tissue homogenates were mixed with a 2 × solution of sample buffer (62.5 mM Tris–HCl, pH 6.8, containing 2% (w/v) SDS, 25% (w/v) glycerol, 5% (v/v) β-mercaptoethanol, and 0.01% (w/v) bromophenol blue) and heated at 95 °C for 3 min. Next, 40 μg of protein sample was subjected to sodium dodecyl sulfate polyacrylamide gel electrophoresis (SDS-PAGE) and transferred onto polyvinylidene difluoride (PVDF) membranes (Millipore-Linco, St Charles, MO). Membranes were blotted for 1 h in 5% (w/v) fat free milk and incubated overnight at 4 °C with primary antibodies against eNOS (1:1500, ab5589), p-eNOS (1:1000, ab184154), PKG1 (1:2000, ab90787, Abcam, Cambridge, UK), and β-actin (1:3000, Bioworld Technology, Nanjing, China). The bound antibody was detected using a horseradish peroxidase (HRP)-conjugated secondary antibody at room temperature for 1 h. Bands were visualized by enhanced chemiluminescence (ECL, Millipore-Linco, St. Charles, MO) and analyzed using ImageJ (National Institutes of Health, Bethesda, Maryland, USA). All protein expression was normalized to that of β-actin, which served as an internal control.

### Morphologic analysis and immunofluorescence

Hematoxylin–eosin (HE) and staining Masson’s trichrome staining were performed to evaluate general morphology and collagen formation by conventional methods. The free wall of the LV was divided into three layers(subendocardium, mid-wall, and subepicardium), which were cut at 10 μm thickness parallel to the apical-basal axis for Masson’s trichrome and perpendicular to the apical-basal axis for TH immunofluorescence staining. The aorta was cut perpendicular to its long axis for TH immunofluorescence staining. We also cut the LV subendocardium and LA tissue perpendicular to the cardiomyocyte long axis for HE staining to assess the cross-sectional area (CSA) of cardiomyocytes. Five randomly selected fields of each section were assessed in a blinded fashion and one representative image was selected.

For TH immunofluorescence staining, tissue sections were deparaffinized, rehydrated, antigen retrieved and blocked in 10% normal goat serum (Vector Laboratories, Burlingane, CA, USA), followed by incubation with a primary antibody against TH (1:200, AB152, Millipore, Temecula, CA, USA) overnight at 4 °C. The bound antibodies were detected with Alexa Fluor-conjugated secondary antibodies (1:1000, Invitrogen, Carlsbad, CA, USA). The fluorescence images were obtained using a fluorescence microscope (Nikon Eclipse E800, Tokyo, Japan) at excitation and emission wavelengths of 543 nm and 573 nm, respectively, and measured with Axiovision software (Zeiss, GmBH, Oberkochen, Germany).

### Statistical analysis

All data are expressed as the mean ± standard deviation (SD). We employed one-way analysis of variance (ANOVA) or two-way repeated measures ANOVA followed by the Bonferroni post hoc test. SPSS 22.0 (SPSS, Inc., Chicago, IL, USA) was used for the statistical analyses. A p value < 0.05 was deemed statistically significant.

## Results

### Effects of dapagliflozin on cardiovascular biochemical indicators in pigs

As shown in Table 1, a significant difference in pig body weight was observed between the HFpEF and DAPA groups, both of which were significantly greater than that in the Normal group at the 18th week. At the 18th week, TC, LDL and TG were markedly elevated in the HFpEF group when compared to the Normal group, and no significant difference was found after 9 weeks of dapagliflozin treatment. There was no significant difference in HbA1c among the three groups throughout the study, indicating that our HFpEF pig model was free of diabetes. However, glucose excretion in 24-h period was substantially higher in dapagliflozin treated pigs than that in the Normal and HFpEF group. Similar change pattern was found in renal Na^+^ excretion. As expected, the plasma BNP, E, NE and Ang II concentrations in HFpEF pigs were strongly increased when compared with the Normal group. Moreover, the plasma levels of E and NE were significantly reduced in the DAPA group. Furthermore, we measured the NE concentration in LV subendocardium, LA, aorta and renal tissues. Similarly, in the HFpEF group, NE was significantly increased as compared with the Normal group, but more importantly, dapagliflozin treatment significantly decreased the NE concentration in the aortic tissues of HFpEF pigs. Data of plasma measurement results from baseline and from the 9th week are presented in Additional file 2: Table S2. Additional data of renal excretion of electrolyte, osmolality and uric acid concentrations in pigs at the 18th week are are presented in Additional file 2: Table S3.

**Table 1:** Characteristics and biochemical indicators of pigs at the 18th week

	Normal	HFpEF	DAPA
n	10	10	10
BW (kg)	63.3 ± 7.2	79.6 ± 5.4^a^	69.7 ± 6.5^a,b^
HR (bpm)	74.2 ± 9.6	61.3 ± 7.8	75.9 ± 5.3
*Plasma biochemistry*			
TC (mg/dl)	67.4 ± 3.3	543.5 ± 54.9^a^	515.7 ± 49.7^a^
LDL (mg/dl)	23.5 ± 3.1	352.7 ± 32.8^a^	375.8 ± 26.8^a^
HDL (mg/dl)	35.7 ± 2.3	196.4 ± 23.1^a^	180.7 ± 28.3^a^
TG (mg/dl)	28.6 ± 6.8	54.7 ± 13.1^a^	59.6 ± 12.4^a^
HbA1c (%)	5.3 ± 1.2	5.6 ± 0.8	5.5 ± 1.1
*Urine biochemistry*			
Glucose (g/24 h)	0.1 ± 0.01	0.1 ± 0.01	24.0 ± 2.1^a,b^
Na^+^ (mmol/24 h)	63.0 ± 5.4	54.6 ± 5.1^a^	93.4 ± 8.5^a,b^
*Plasma ELISA* (pg/ml)			
BNP	56.9 ± 14.5	76.2 ± 15.3^a^	77.3 ± 10.7^a^
E	76.9 ± 14.5	125.2 ± 17.3^a^	113.2 ± 28.7^a,b^
NE	60.7 ± 14.3	179.6 ± 17.3^a^	163.8 ± 20.9^a,b^
Ang II	200.8 ± 19.7	476.5 ± 26.3^a^	467.4 ± 33.1^a^
*Tissue ELISA (NE)* (pg/ug)			
LA	11.6 ± 4.2	19.8 ± 5.9^a^	18.4 ± 3.5^a^
LV	15.1 ± 3.6	29.7 ± 3.8^a^	27.5 ± 8.7^a^
Aorta	25.6 ± 7.9	71.4 ± 13.5^a^	55.6 ± 11.9^a,b^
Kidney	8.2 ± 3.1	16.3 ± 4.7^a^	18.9 ± 3.0^a^

Values are expressed as the mean ± SD. Statistical analyses were performed by one-way ANOVA followed by the Bonferroni post hoc test

^a^*p *< 0.05 vs. the Normal group

^b^*p *< 0.05 vs. the HFpEF group

### Dapagliflozin treatment improved high blood pressure in pigs with HFpEF

**Fig. 2:**
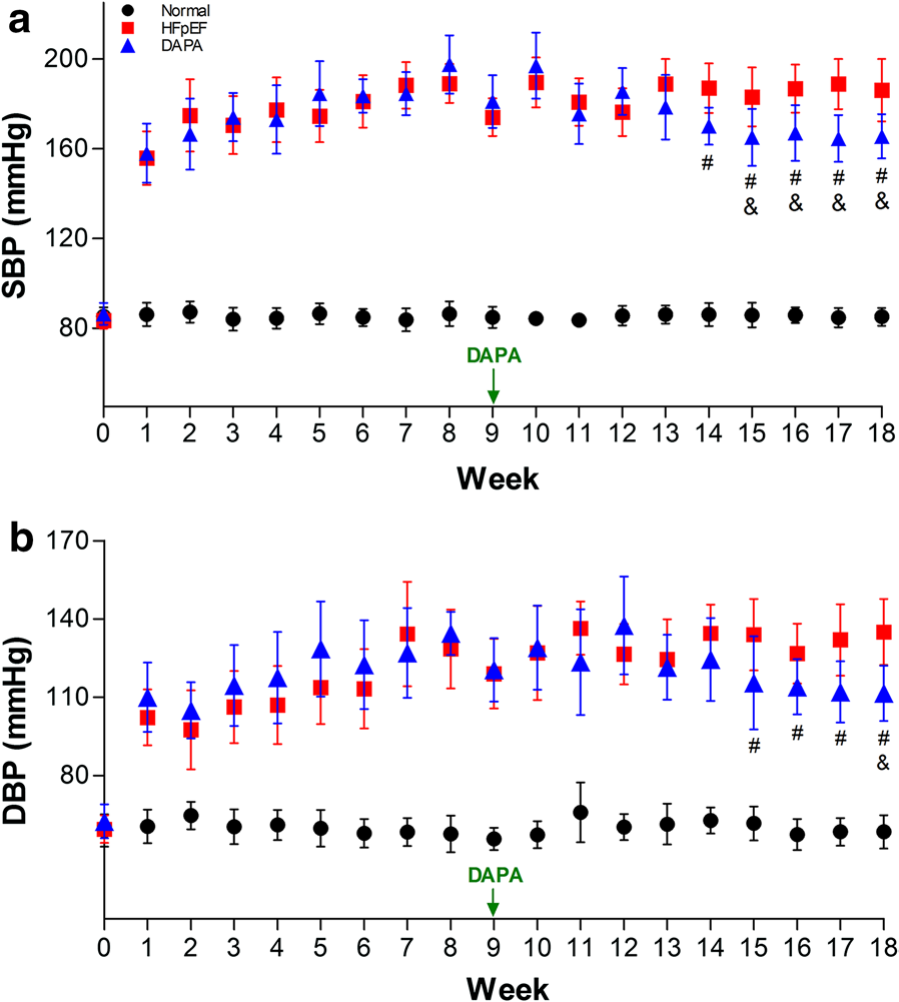
Serial measurements of blood pressure in pigs. The blood pressure of experimental pigs was measured weekly. SBP (**a**): systolic blood pressure; DBP (**b**): diastolic blood pressure. The data are presented as the mean ± SD. n = 10 pigs per group. Two-way repeated measures ANOVA was performed. ^#^*p *< 0.05 vs. the HFpEF group, ^&^*p *< 0.05 vs. the 9th week of the same group

During the study, the blood pressure of experimental pigs was measured weekly. After 1 week of dapagliflozin administration, both the systolic (Fig. 2a) and diastolic (Fig. 2b) blood pressure in the HFpEF and DAPA groups were significantly and continuously higher than those in the Normal group until the end of the study (all *p *< 0.01). Importantly, the SBP was significantly lower in the DAPA group than in the HFpEF group from the 14th to the 18th week, while the DBP showed comparable results from the 15th to the 18th week. In the DAPA group, when comparing the blood pressure in the 18th week to that in the 9th week, both SBP and DBP were decreased after dapagliflozin treatment, suggesting that dapagliflozin could prevent the development of hypertension in HFpEF pigs.

### Dapagliflozin alleviated heart concentric remodeling and partly prevented LV fibrosis in HFpEF pigs

**Fig. 3:**
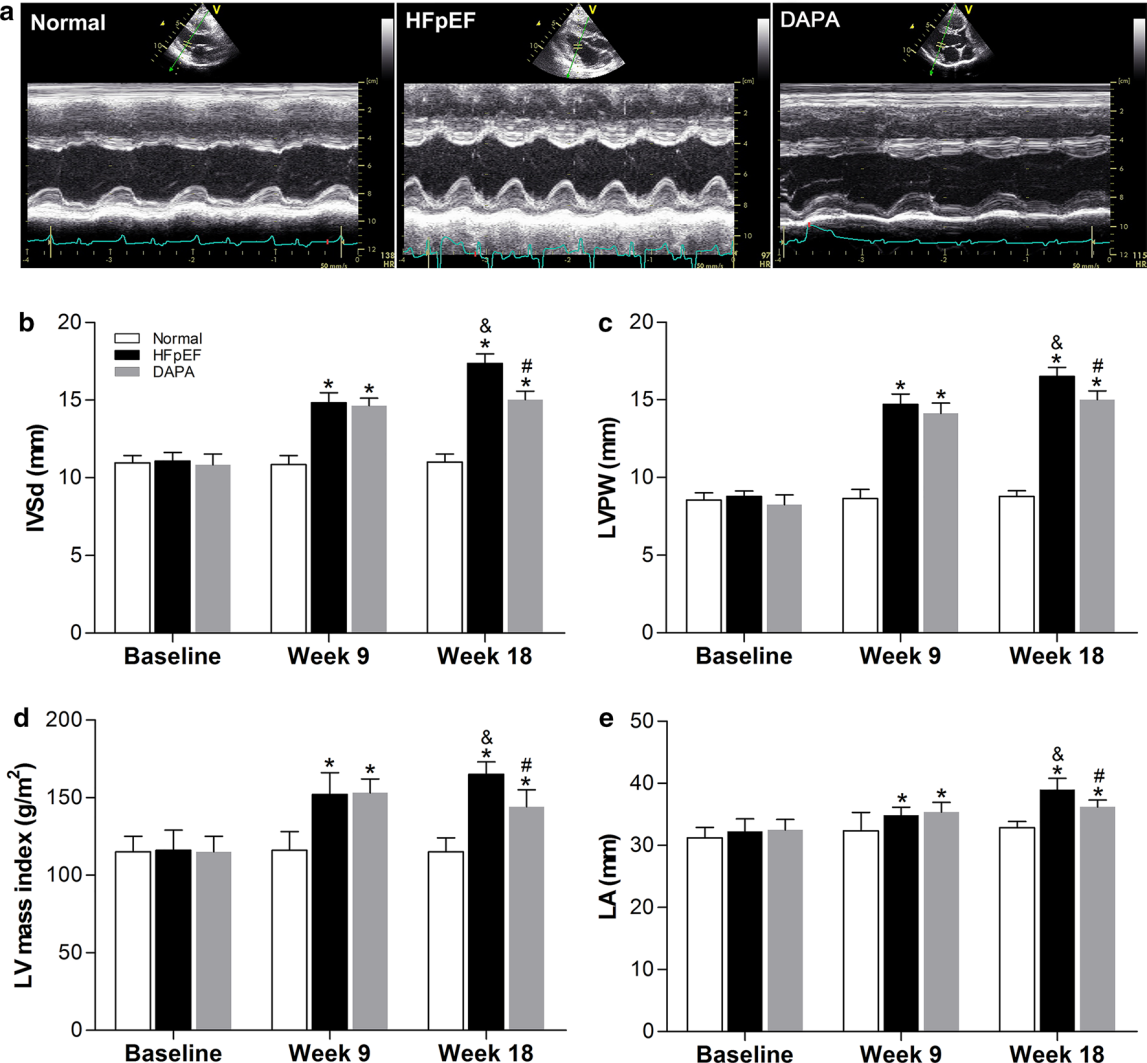
Dapagliflozin alleviated heart concentric remodeling in HFpEF pigs. At the indicated time points, structural changes in the heart were measured by echocardiography. Representative images of echocardiographic M-mode Doppler imaging are shown (**a**); IVSd (**b**): interventricular septum thickness at end-diastole; LVPW (**c**): left ventricular posterior wall; LV mass index (**d**); LA (**e**): left atrium. The data are presented as the mean ± SD. n = 10 pigs per group. Two-way repeated measures ANOVA was performed. ^*^p < 0.05 vs. the Normal group, ^#^*p *< 0.05 vs. the HFpEF group, ^&^*p *< 0.05 vs. the 9th week of the same group

To explore structural alterations in the left heart, several key parameters were measured by echocardiography. Representative images of echocardiographic M-mode Doppler imaging are shown (Fig. 3a). Pigs in the HFpEF group exhibited a significant increase in the thickness of the end-diastolic ventricular septum (IVSd), LV posterior wall (LVPW), LV mass index and left atrium dimension (LA) compared with the Normal group at the 9th week, and the difference was more obvious at the end of the study (Fig. 3b–e). These structural alterations in the heart confirmed the characteristics of HFpEF in our pig model. Notably, significant decreases in these cardiac structure remodeling parameters were detected after 9 weeks of dapagliflozin treatment in the DAPA group, indicating the effect of dapagliflozin on mitigating LV concentric remodeling and LA enlargement in HFpEF (Fig. 3b–e).

**Fig. 4:**
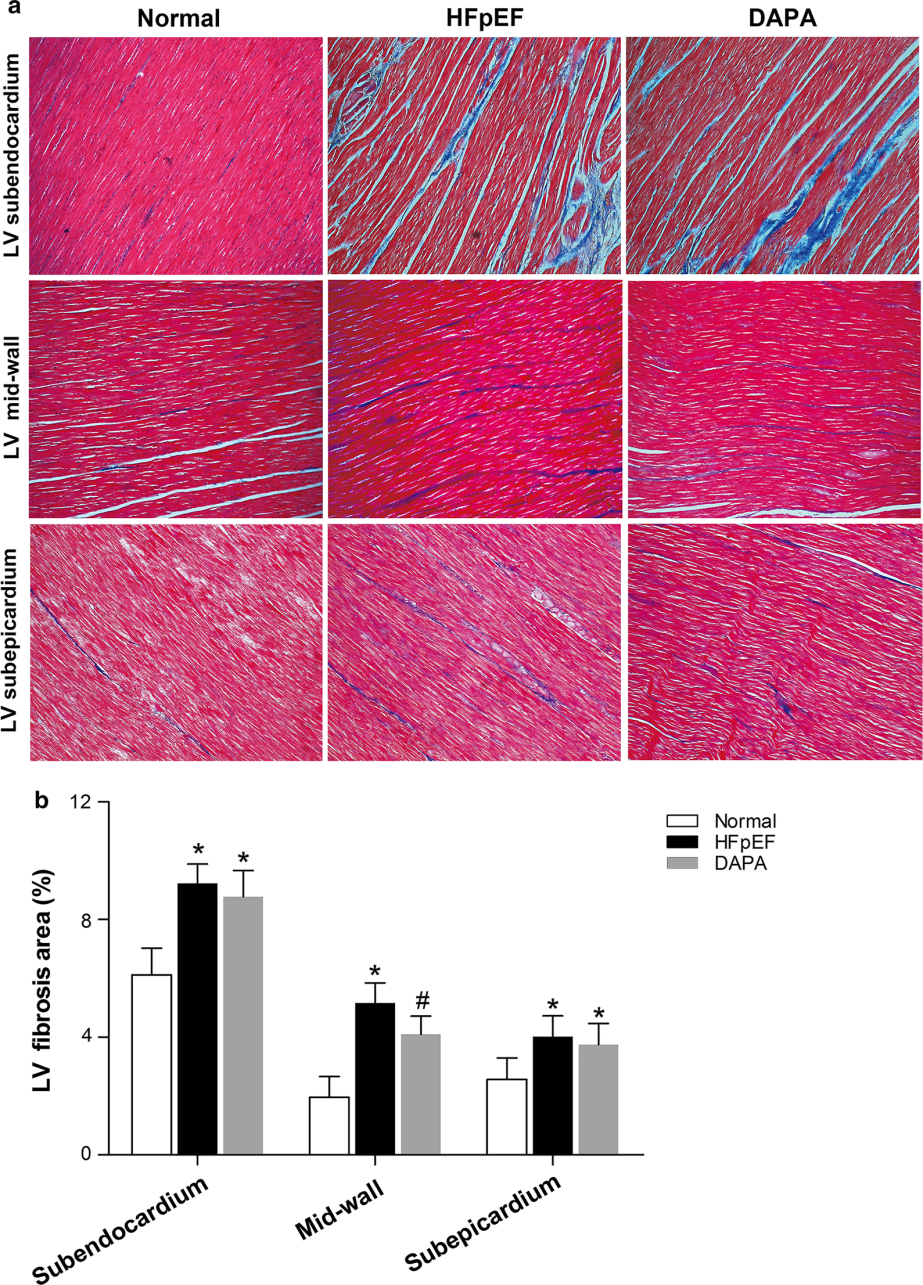
Effects of dapagliflozin treatment on LV fibrosis in HFpEF pigs. Representative images of Masson’s trichrome staining are shown (**a**). The area percentages of fibrosis were calculated (**b**). Values are expressed as the mean ± SD. n = 10 pigs per group. Statistical analyses were performed by one-way ANOVA followed by the Bonferroni post hoc test. ^*^p < 0.05 vs. the Normal group, ^#^*p *< 0.05 vs. the HEpEF group

To further investigate LV fibrosis in HFpEF pigs and the effect of dapagliflozin, Masson’s trichrome staining was performed. As shown in Fig. 4, HFpEF caused a marked increase in collagen deposits, predominantly in the subendocardial layer, as well as in the mid-wall and subepicardium layers of the LV. However, there was a modest amelioration in LV fibrosis in the DAPA group, but the change in only the mid-wall layer was significant. Similarly, we also found that both LV and LA cardiomyocyte CSA and LA fibrosis were strongly increased in the HFpEF group compared to the Normal group. However, no significant change was observed after 9 weeks of dapagliflozin treatment (Additional file 4: Fig. S1).

### Dapagliflozin modulated LV contractility and pulmonary artery pressure in HFpEF pigs

**Fig. 5:**
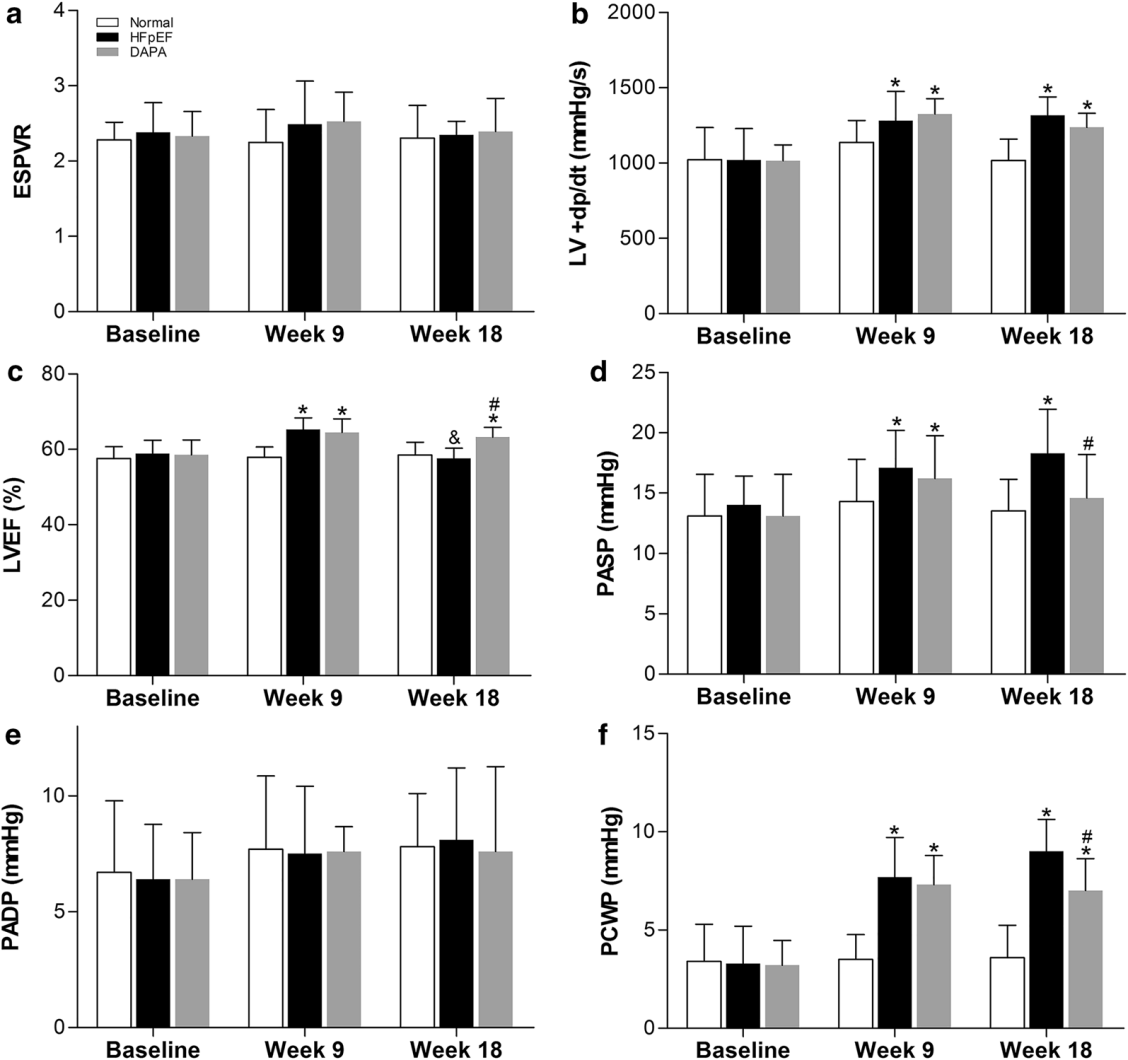
Serial measurements of hemodynamic parameters in pigs. Invasive cardiac catheterization was conducted to determine LV contractility and alterations in pulmonary pressure. ESPVR (**a**): end-systolic pressure–volume relationship. LV + dp/dt (**b**): the rate of LV pressure rise in early systole; LVEF (**c**): left ventricular ejection fraction; PASP (**d**): pulmonary artery systolic pressure; PADP (**e**): pulmonary artery diastolic pressure; PCWP (**f**): pulmonary capillary wedge pressure; Values are expressed as the mean ± SD. n = 10 pigs per group. Two-way repeated measures ANOVA was performed. ^*^p < 0.05 vs. the Normal group, ^#^*p *< 0.05 vs. the HFpEF group, ^&^*p *< 0.05 vs. the 9th week of the same group

Invasive left cardiac catheterization was carried out to determine LV contractility by calculating the values of ESPVR, LV + dp/dt and LVEF. As depicted in Fig. 5, ESPVR remained unchanged throughout the whole study in the three groups of pigs (Fig. 5a). In the HFpEF and DAPA groups, LV + dp/dt and LVEF were modestly elevated compared with the Normal group at the 9th and 18th weeks (Fig. 5b, c). Furthermore, at the end of the study, dapagliflozin even led to a significant improvement in LVEF when compared with the HFpEF group. Similarly, the CO of pigs remained unchanged throughout the whole study in the three groups (Additional file 5: Fig. S2A). These results verified the normal LV systolic function and preserved ejection fraction (EF) in our pig model.

Additionally, invasive right cardiac catheterization was performed to investigate the alteration in pulmonary pressure under the conditions of HFpEF and dapagliflozin treatment. PASP and PCWP were significantly higher in HFpEF pigs compared with the Normal group, especially at the 18th week. Interestingly, both of PASP and PCWP them were significantly decreased after 9 weeks of dapagliflozin treatment (Fig. 5d, f). Therefore, for the first time, we found that dapagliflozin may improve pulmonary hypertension that develops in HFpEF pigs.

### Effects of dapagliflozin on LV diastolic function and the compliance of HFpEF pigs

**Fig. 6:**
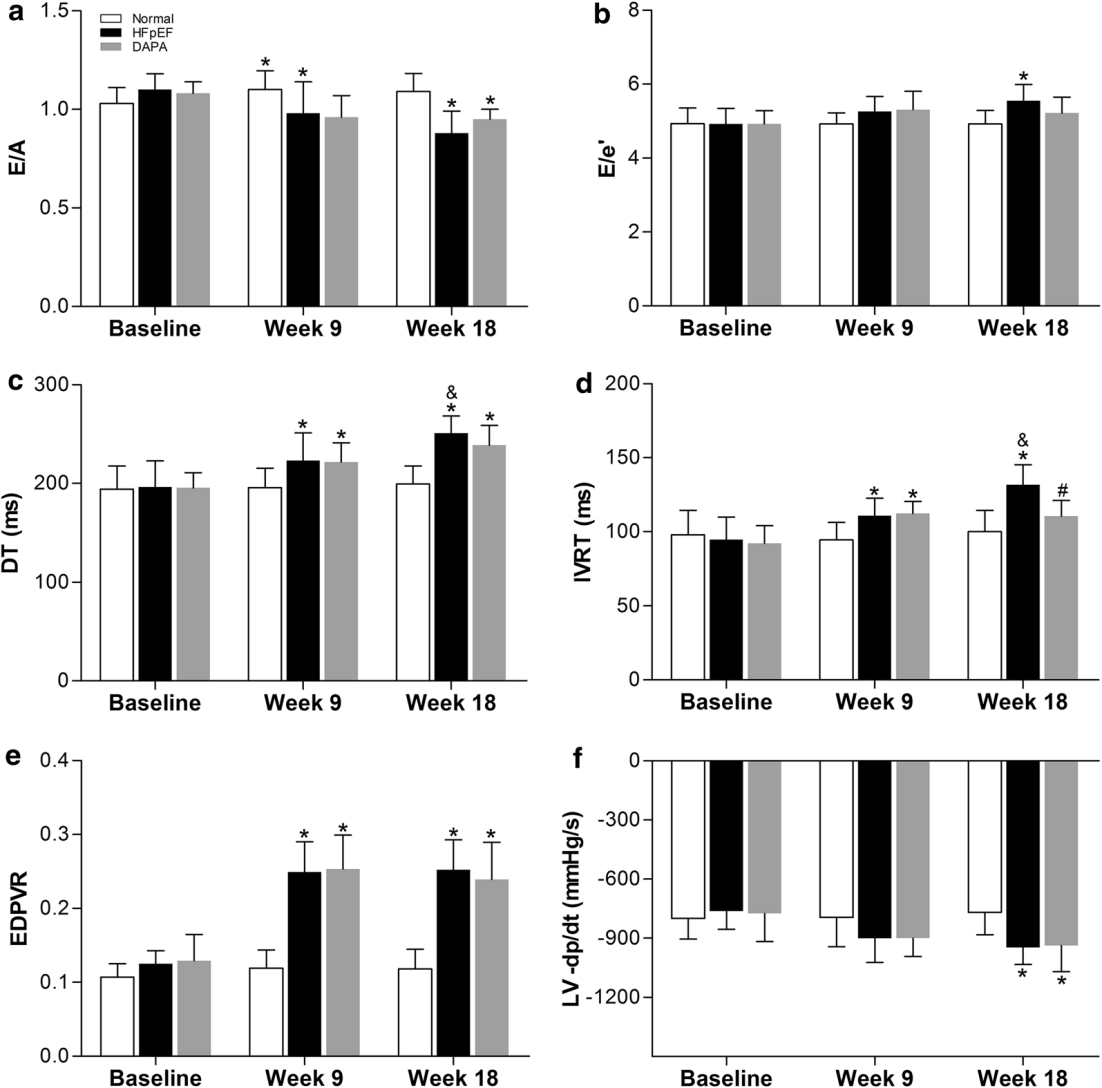
Effects of dapagliflozin on LV diastolic function and the compliance of HFpEF pigs. The functional status of the LV in HFpEF pigs with or without dapagliflozin treatment was assessed by echocardiography. E/A (**a**); E/e′ (**b**); DT (**c**): deceleration time; IVRT (**d**): isovolumetric relaxation time; EDPVR (**e**): end-diastolic pressure–volume relationship; LV-dp/dt (**f**): the rate of LV pressure decline in early diastole. Data are expressed as the mean ± SD. n = 10 pigs per group. Two-way repeated measures ANOVA was performed. ^*^p < 0.05 vs. the Normal group, ^#^*p *< 0.05 vs. the HFpEF group, ^&^*p *< 0.05 vs. the 9th week of the same group

We measured LV function (especially diastolic function) and compliance by echocardiography and invasive cardiac catheterization. As shown in Fig. 6, compared to the Normal group, representative parameters implicated in LV diastolic function, such as E/e′, DT and IVRT, were significantly increased in HFpEF pigs at the 18th week and accompanied by decreased E/A (Fig. 6a–d), suggesting impaired LV diastolic function in the HFpEF pig model. In contrast, 9 weeks of dapagliflozin treatment modestly prevented these changes, and the decrease in IVRT was statistically significant. Since EDPVR and LV-dp/dt are commonly used indicators in monitoring LV diastolic function in HFpEF patients [24, 25], we further measured EDPVR and LV-dp/dt via invasive left cardiac catheterization, and found an evident increase in HFpEF pigs compared with the Normal group, indicating a loss of LV compliance and increased LV filling pressure. Nevertheless, the effect of dapagliflozin treatment on both indexes did not achieve statistical significance (Fig. 6e, f). We also measured the LV diameter at the end of diastole (LVEDD) and systole (LVESD); however, no noticeable changes were discovered among the three groups at the indicated time points (Additional file 5: Fig. S2B, C).

### Dapagliflozin prevented aortal sympathetic tone and stiffness in HFpEF pigs

**Fig. 7:**
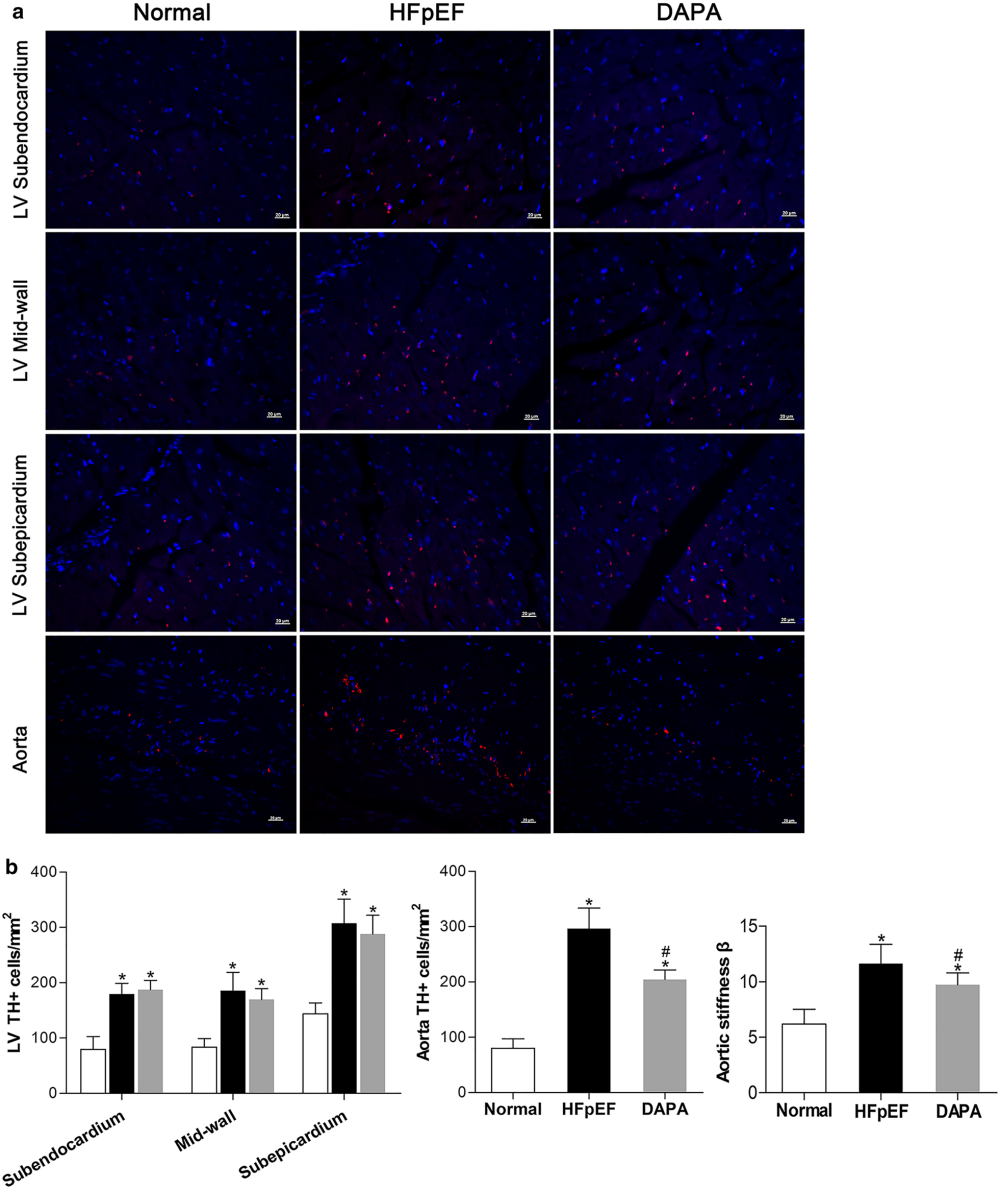
Dapagliflozin prevented aortal sympathetic tone and stiffness in HFpEF pigs. TH expression in tissues from the LV and aorta was determined by immunofluorescence staining at the end of the study. Representative images are shown (**a**), TH-positive cells in the CSA of the LV and aorta and aortic stiffness index β were calculated (**b**). Values are expressed as the mean ± SD. n = 10 pigs per group. Statistical analyses were performed by one-way ANOVA followed by the Bonferroni post hoc test. ^*^p < 0.05 vs. the Normal group, ^#^*p *< 0.05 vs. the HEpEF group

As TH is a pivotal enzyme involved in catecholamine synthesis and usually reflects sympathetic tone, we measured TH expression in tissues from the LV and aorta by immunofluorescence staining. As shown in Fig. 7, TH-positive cells in the LV subendocardium, mid-wall and subepicardium layers of HFpEF pigs were significantly increased when compared with the Normal group. However, dapagliflozin had no significant impact on TH expression in the LV, indicative of unchanged left ventricular sympathetic tension. Remarkably, enhanced TH expression in the aortas of HFpEF pigs was strongly prevented by dapagliflozin treatment, suggesting a downregulating effect of dapagliflozin on sympathetic tone in the aorta. This compelling finding may help us understand the antihypertensive effect of dapagliflozin in the context of HFpEF. As expected, the aortic stiffness β was significantly higher in HFpEF pigs than in Normal pigs at the 18th week. However, treatment with dapagliflozin for 9 weeks could effectively improve aortic stiffness under HFpEF conditions (Fig. 7b). Data of aortic stiffness β calculated from baseline and from the 9th week are listed in Additional file 2: Table S2.

### Dapagliflozin inhibited inflammation and activated the NO-cGMP-PKG pathway in the aorta

**Fig. 8:**
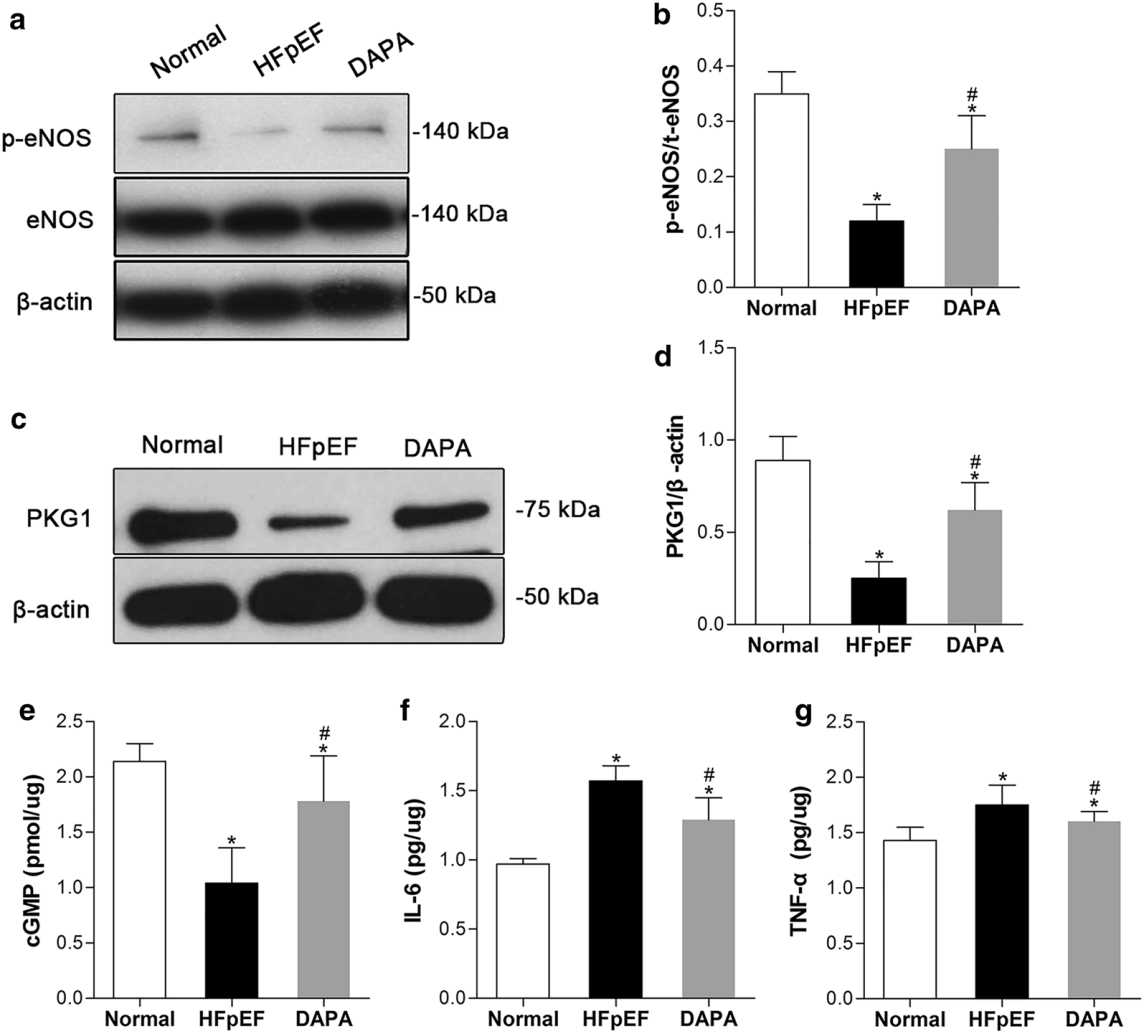
Beneficial effects of dapagliflozin on inflammation and the NO-cGMP-PKG pathway in the aorta. Aortic signaling pathway proteins (p-eNOS, NOS and PKG1) were determined by Western blotting (**a**, **c**), and the relative protein levels were quantified densitometrically with ImageJ (**b**, **d**). Available ELISA kits were used to measure the levels of aortic cGMP (**e**) and proinflammatory cytokines (IL-6 and TNF-α) (**f**, **g**). β-Actin was used as an internal control. Values are shown as the mean ± SD. ^*^p < 0.05 vs. the Normal group, ^#^*p *< 0.05 vs. the HEpEF group

The NO-cGMP-PKG signaling pathway in endothelium cells is postulated to be one of the most important factors associated with vasodilation, resulting in a reduction in blood pressure. As presented in Fig. 8a–e, we found that the aortic NO-cGMP-PKG signaling pathway was suppressed in pigs with HFpEF, as evidenced by reduced cGMP levels in the aorta and the downregulated protein expression of PKG1 and phosphorylated eNOS. Interestingly, dapagliflozin substantially restored the activity of the NO-cGMP-PKG pathway by promoting the phosphorylation of eNOS and subsequently increased the levels of cGMP and PKG1 in aortic tissue. As expected, the levels of the inflammatory cytokines IL-6 and TNF-α were elevated in the aortas of HFpEF pigs and significantly decreased with dapagliflozin treatment (Fig. 8f, g), suggesting that dapagliflozin could attenuate the hypertension-induced macrovascular inflammatory response.

## Discussion

The data in the current study provide a deeper understanding of cardiac structure remodeling, cardiac function and sympathetic tone alterations, and the molecular mechanism involved in HFpEF. With regard to the therapeutic effect of dapagliflozin on HFpEF, the following issues were addressed in this work: (1) Dapagliflozin treatment could decrease both SBP and DBP and prevent the progression of LV concentric hypertrophy remodeling and LA dilation in non-diabetic HFpEF pig models with favorable safety and effectiveness; (2) With echocardiography and an invasive hemodynamic assessment, no discernible effects of 9 weeks of dapagliflozin treatment on LV fibrosis or diastolic function were observed in HFpEF pigs; (3) Dapagliflozin treatment significantly decreased plasma adrenal medullary hormone levels in HFpEF model, and strongly mitigated the sympathetic tension of aortic tissue but not that of the LV myocardium; in the aorta, the inflammatory response and the NO-cGMP-PKG signaling pathway were deteriorated, and both were improved by dapagliflozin treatment; and (4) It is pertinent to note that dapagliflozin presented a negative regulatory effect on PASP and PCWP.

Dapagliflozin is a newly developed oral antidiabetic drug that enhances renal glucose excretion or glycosuria and reduces hyperglycemia by the highly selective inhibition of SGLT2 [11]. Previous studies have observed the effect of SGLT2 inhibitors on decreasing blood pressure in patients with T2DM [26–28], consistent with our results obtained from 9 weeks of dapagliflozin treatment in HFpEF pigs. The mechanisms of BP reduction with SGLT2 inhibitors have not been fully elucidated but likely involve several pathways, including a modest diuretic effect, weight reduction and potentially sodium depletion [29]. More intriguingly, data obtained following 2-day treatment with dapagliflozin in T2DM patients demonstrated that acute treatment with dapagliflozin significantly improved systemic endothelial function, arterial stiffness and the renal resistive index [30]. Arteriole remodeling that was observed in T2DM patients was alleviated after dapagliflozin treatment [13]. In our study, as presented with TH immunofluorescence staining and the ELISA results of NE and E in the cardiovascular system, sympathetic tone was considerably enhanced in HFpEF pigs. However, we found that treatment with dapagliflozin significantly decreased plasma adrenal medullary hormone levels in HFpEF models and strongly mitigated the sympathetic tension of aortic tissue. Thus, a direct vascular effect of dapagliflozin might also contribute to BP changes.

Dapagliflozin was also found to improve arterial stiffness, endothelial dysfunction and vascular smooth muscle dysfunction and reduce circulating markers of inflammation in type 2 diabetic mice [18], which are supported by our results from the HFpEF pig model without diabetes. NO-cGMP-PKG signaling is an essential pathway for vascular dilation and results in a lowering of blood pressure by decreasing Ca^2+^ sensitization and/or activating Ca^2+^-activated K^+^ channels to reduce the intracellular Ca^2+^ concentration [31, 32]. Macrovascular inflammation cytokines such as IL-6 and TNF-α can reduce the bioavailability of NO, which is generated by constitutive eNOS in endothelial cells. Previously, Paulus et al. [8] described a novel paradigm for HFpEF in which coronary microvascular inflammation and deficient NO-cGMP-PKG signaling in the myocardium are responsible for diastolic LV stiffness and HFpEF development, which was confirmed in subsequent studies [9, 10]. For the first time, we observed an aggravated inflammatory response and inhibition of the NO-cGMP-PKG signaling pathway in aortas of HFpEF pigs, both of which were improved by dapagliflozin treatment. In addition, previous studies have demonstrated that enhanced sympathetic tone was implicated in the development of systematic inflammation, and we found that dapagliflozin treatment was able to alleviate sympathetic tension in the aortas of HFpEF pigs. Moreover, the change in sympathetic tension in the aorta was exactly concordant with that of the inflammation status and NO-cGMP-PKG pathway activity in dapagliflozin-treated HFpEF pigs. Thus, dapagliflozin may emerge as a novel therapy to normalize high blood pressure by easing macrovascular sympathetic stress and the downstream inflammation-NO-cGMP-PKG pathway. Notably, although metabolic comorbidities attendant upon HFpEF, such as overweight/obesity and hyperlipidemia, can usually cause chronic systemic inflammation [8], no significant effect of dapagliflozin treatment on the lipid profile of HFpEF pigs was observed. Similarly, Gian et al. [33] also found that therapy with dapagliflozin exerted no significant effect on HDL cholesterol levels or HDL functionality. However, whether dapagliflozin treatment effects other aspects of systemic metabolism should be further explored.

Schwarzl reported a HFpEF model induced by DOCA (100 mg/kg, 90-day release subcutaneous depot) and a WD containing high content of salt (4%), cholesterol (4%), crude fat (25%), cholate (0.5%), and sugar (26%) for 12 weeks in landrace pigs [6]. On this basis, we added angiotension II infusion, which was thought to be a crucial factor in the development of chronic HF from the results of clinical trials and animal studies [34]. In addition, the WD used in our model did not contain extra added sugar. As expected, we created a valid HFpEF pig model within a shorter experimental period (3 weeks ahead of the method Schwarzl reported). Blood pressure was consistently higher than that of Schwarzl’s model from the first week of study. Notably, our HFpEF pigs did not develop diabetes, however, neither blood glucose nor HbA1c was not determined in their study. Specific LV remodeling, characterized by significant concentric LV hypertrophy and myocardial fibrosis, was found in HFpEF pigs that ultimately led to a loss of LV compliance and increased LV filling pressures, i.e., a leftward shift of the LV end-diastolic pressure–volume relationship (EDPVR) [6]. The above characteristics are fully reflected in our HFpEF pig model, indicating that the establishment of the HFpEF pig model was successful. Swine and human subjects have similarities in their cardiovascular system and share many characteristics of excitation–contraction coupling [7]. In addition, SGLT2i has been shown to improve HFrEF in non-diabetic pig models [35], which is the foundation for clinical trials investigating the efficacy of SGLT2i for HF in non-diabetic patients [36]. Soga et al. [37] reported the beneficial effect of dapagliflozin on LV diastolic function in T2DM patients with HF (HFpEF, HFrEF or HFmrEF), but no significant improvements in blood pressure, HbA1c and lipid profiles were observed, indicating the complex mechanisms of SGLT2i action on HF. We were the first to develop a relevant, risk factor-based porcine model characterized by hypertension and hyperlipidemia, not diabetes, to explore effects and potential mechanisms of dapagliflozin on the cardiovascular system in conditions of HFpEF. In the present study, the echocardiography results revealed that concentric LV hypertrophy was reversed by dapagliflozin treatment, which is very likely to benefit from the systemic blood pressure drop. Additionally, myocardial fibrosis deposition and diastolic dysfunction were significantly exacerbated in the HFpEF group and ameliorated with 9 weeks of dapagliflozin treatment without statistical significance, which was not consistent with previous studies [25, 38]. However, owing to the diabetic models used and the long experimental period in those studies, the therapeutic effect of an SGLT2i can completely emerge. Due to the rapid growth of pigs, our overall experimental period was limited to 18 weeks and included only 9 weeks of dapagliflozin treatment. Thus, we may speculate that the results of the present study represent an initial effect of dapagliflozin on HFpEF. If the treatment period of dapagliflozin is extended, the therapeutic effect would be more remarkable.

Furthermore, we noted that the response of TH expression to dapagliflozin treatment was more sensitive in the aorta than in the LV. The underlying mechanism of this disparity remains elusive. We speculate that the experimental period and the plasma concentration of dapagliflozin should be considered. Owing to the limited data on dapagliflozin in the HFpEF pig model, more work in this area is sorely needed. In addition, we found that LA was dilated, and thus, PASP and PCWP were increased in HFpEF pigs, consistent with prior studies. Interestingly, the effect of dapagliflozin on attenuating the detrimental PASP and PCWP in HFpEF pigs was significant [39, 40]. Since PASP is closely linked to increased morbidity and mortality in HFpEF patients, based on our findings, dapagliflozin could be a potential strategy to improve pulmonary hypertension and provide benefits for HFpEF patients [40]. However, the detailed mechanisms whereby dapagliflozin decreases PASP and PCWP are yet poorly understood.

## Conclusion

The results presented here demonstrate that dapagliflozin might decrease blood pressure and prevent the progression of LV concentric hypertrophy in the HFpEF pig model by mitigating sympathetic nerve tone and the inflammatory response in the aorta, leading to reactivation of the NO-cGMP-PKG pathway. These data are important in elucidating the potential mechanisms of the dapagliflozin-mediated improvement in cardiac function and provide a basis for the further development of innovative therapeutic strategies.

## Supplementary information

**Additional file 1: Table S1.** Detailed ingredient contents of Standard diet and Western diet.

**Additional file 2: Table S2.** Characteristics and biochemical indicators of pigs at baseline and at the 9th week. Values are expressed as the mean ± SD. Statistical analyses were performed by one-way ANOVA followed by the Bonferroni post hoc test. ^a^*p* < 0.05 vs. the Normal group at the same time point.

**Additional file 3: Table S3.** Data of renal excretion in pigs at the 18th week. Values are expressed as the mean ± SD. Statistical analyses were performed by one-way ANOVA followed by the Bonferroni post hoc test. ^a^*p* < 0.05 vs. the Normal group, ^b^*p* < 0.05 vs. the HFpEF group.

**Additional file 4: Fig. S1.** Dapagliflozin had no significant effect on cardiomyocyte CSA or LA fibrosis in HFpEF pigs. The cardiomyocyte CSA of the LV and LA was calculated (A and B). Representative images of Masson’s trichrome staining of the LA posterior wall are shown (C). The area percentages of LA fibrosis were calculated (D). Values are expressed as the mean ± SD. n = 10 pigs per group. Statistical analyses were performed by one-way ANOVA followed by the Bonferroni post hoc test. ^*^p < 0.05 vs. the Normal group.

**Additional file 5: Fig. S2.** Dapagliflozin had no significant effect on CO, LVEDD or LVESD in HFpEF pigs. CO, cardiac output; LVEDD, left ventricular end-diastolic dimension; LVESD, left ventricular end-systolic dimension. Values are expressed as the mean ± SD. n = 10 pigs per group. Statistical analyses were performed by one-way ANOVA followed by the Bonferroni post hoc test. ^*^p < 0.05 vs. the Normal group.

## Data Availability

The datasets generated and analyzed for this study are available from the corresponding author upon reasonable request.

## Abbreviations

HF: heart failure
HEpEF: heart failure with preserved ejection fraction
HFrEF: heart failure with reduced ejection fraction
HFmrEF: heart failure with mid-range ejection fraction
T2DM: type 2 diabetes mellitus
SGLT2i: sodium-glucose cotransporter 2 inhibitor
LVEF: left ventricle ejection fraction
DOCA: deoxycorticosterone acetate
LA: left atrium
LV: left ventricle
SBP: systolic blood pressure
DBP: diastolic blood pressure
IVSd: interventricular septum at end-diastole
LVPW: left ventricular posterior wall
DT: deceleration time
IVRT: isovolumetric relaxation time
ESPVR: end-systolic pressure–volume relationship
EDPVR: end-diastolic pressure–volume relationship
PASP: pulmonary artery systolic pressure
PADP: pulmonary artery diastolic pressure
PCWP: pulmonary capillary wedge pressure
TH: tyrosine hydroxylase
BNP: brain natriuretic peptide
E: epinephrine
NE: norepinephrine
Ang II: angiotensin II
eNOS: endothelial nitric oxide synthase
cGMP: cyclic guanosine monophosphate
PKG: protein kinase G
TC: total cholesterol
TG: triglyceride
LDL: low-density lipoprotein
HDL: high-density lipoprotein
CSA: cross-sectional area
