# Supplementary material for: Dapagliflozin improves left ventricular remodeling and aorta sympathetic tone in a pig model of heart failure with preserved ejection fraction
Source: Cardiovasc Diabetol. 2019 Aug 20;18:107. doi: 10.1186/s12933-019-0914-1 (PMC6702744; doi:10.1186/s12933-019-0914-1)
Supplement: Supplementary file 1 — Additional file 1: Table S1. Detailed ingredient contents of Standard diet and Western diet. [file 12933_2019_914_MOESM1_ESM.docx]

| Ingredient | Proportion of total calories | |
| --- | --- | --- |
|  | Standard diet (n=10) | Western diet (n=20) |
| Carbohydrates (%) | 71.0 | 40.5 |
| Protein (%) | 18.5 | 19.5 |
| Fat (%) | 10.5 | 40.0 |
| *Fat source* | *Hydrogenated soybean oil* | *Hydrogenated soybean oil* |
| Added Cholesterol (%) | 0 | 4% by weight |
| Added Sodium Cholate (%) | 0 | 0.7% by weight |
| Added Salt (%) | 0 | 2% by weight |
| Added Sugar (%) | 0 | 0 |

**Table S1.** **Detailed ingredient contents of Standard diet and Western diet.**
