# Supplementary material for: Dapagliflozin improves left ventricular remodeling and aorta sympathetic tone in a pig model of heart failure with preserved ejection fraction
Source: Cardiovasc Diabetol. 2019 Aug 20;18:107. doi: 10.1186/s12933-019-0914-1 (PMC6702744; doi:10.1186/s12933-019-0914-1)
Supplement: Supplementary file 2 — Additional file 2: Table S2. Characteristics and biochemical indicators of pigs at baseline and at the 9th week. Values are expressed as the mean ± SD. Statistical analyses were performed by one-way ANOVA followed by the Bonferroni post hoc test. ap < 0.05 vs. the Normal group at the same time point. [file 12933_2019_914_MOESM2_ESM.docx]

|  | **Normal (n=10)** | | **HFpEF (n=10)** | | **DAPA (n=10)** | |
| --- | --- | --- | --- | --- | --- | --- |
|  | Baseline | 9 weeks | Baseline | 9 weeks | Baseline | 9 weeks |
| BW (kg) | 34.6±2.9 | 50.1±3.5 | 37.1±4.1 | 61.3±5.8^a^ | 35.1±3.3 | 61.8±4.9^a^ |
| HR (bpm) | 74.7±3.8 | 72.6±7.4 | 69.0±5.8 | 77.4±9.2 | 78.4±6.4 | 72.4±7.4 |
| *Plasma biochemistry* | |  |  |  |  |  |
| TC (mg/dl) | 65.3±4.3 | 63.2±2.7 | 65.9±2.7 | 373.5±49.9^a^ | 66.8±5.1 | 358.8±64.7^a^ |
| LDL (mg/dl) | 25.1±4.1 | 26.6±2.4 | 24.6±2.1 | 217.7±29.2^a^ | 23.9±4.2 | 221.3±22.6^a^ |
| HDL (mg/dl) | 36.7±1.9 | 38.0±3.2 | 35.8±3.2 | 100.6±31.2^a^ | 36.3±2.3 | 112.9±25.5^a^ |
| TG (mg/dl) | 27.2±5.4 | 28.9±1.8 | 26.7±5.8 | 42.5±11.8^a^ | 27.8±6.0 | 40.4±14.0^a^ |
| HbA1c (%) | 4.9±1.4 | 5.3±0.9 | 5.0±1.1 | 5.5±1.2 | 4.9±1.0 | 5.4±0.8 |
| *Plasma ELISA* |  |  |  |  |  |  |
| BNP (pg/ml) | 54.5±10.5 | 60.7±7.3 | 55.8±11.3 | 65.9±8.5 | 51.5±14.6 | 60.9±18.5 |
| E (pg/ml) | 84.5±20.5 | 80.7±17.3 | 75.8±11.3 | 85.9±18.7 | 71.5±14.6 | 90.9±18.5 |
| NE (pg/ml) | 59.5±12.1 | 67.4±19.6 | 63.5±14.9 | 127.4±16.5^a^ | 65.8±11.2 | 124.0±17.7^a^ |
| Ang II (pg/ml) | 194.1±13.1 | 210.5±15.3 | 201.4±9.4 | 320.2±26.1^a^ | 205.9±9.8 | 330.7±21 .6^a^ |
| *Aortic stiffness β* | 5.90±1.60 | 5.60±1.33 | 6.20±2.27 | 9.40±2.93^a^ | 6.15±2.40 | 9.85±1.07^a^ |

**Table S2.** **Characteristics** **and biochemical indicators of pigs at baseline and at the 9th week.** Values are expressed as the mean ± SD. Statistical analyses were performed by one-way ANOVA followed by the Bonferroni post hoc test. ^a^*p*<0.05 vs. the Normal group at the same time point.
