# Supplementary material for: Dapagliflozin improves left ventricular remodeling and aorta sympathetic tone in a pig model of heart failure with preserved ejection fraction
Source: Cardiovasc Diabetol. 2019 Aug 20;18:107. doi: 10.1186/s12933-019-0914-1 (PMC6702744; doi:10.1186/s12933-019-0914-1)
Supplement: Supplementary file 3 — Additional file 3: Table S3. Data of renal excretion in pigs at the 18th week. Values are expressed as the mean ± SD. Statistical analyses were performed by one-way ANOVA followed by the Bonferroni post hoc test. ap < 0.05 vs. the Normal group, bp < 0.05 vs. the HFpEF group. [file 12933_2019_914_MOESM3_ESM.docx]

|  | **Normal** | **HFpEF** | **DAPA** |
| --- | --- | --- | --- |
| ***Urine biochemistry*** |  |  |  |
| Volume (ml/24h) | 1756.0±146.0 | 1576.0±159.0^a^ | 2477.0±310.0^a,b^ |
| Osmoles (osm/kg) | 239.0±24.0 | 235.0±21.0 | 296.0±28.0^a,b^ |
| K+ (mmol/24h) | 44.7±3.3 | 40.5±3.6 | 58.8±5.5^a,b^ |
| Urate (mg/24h) | 407.0±19.0 | 416.0±26.0 | 587.0±33.0^a,b^ |

**Table S3.** **Data of renal excretion in pigs at the 18th week.** Values are expressed as the mean ± SD. Statistical analyses were performed by one-way ANOVA followed by the Bonferroni post hoc test. ^a^*p*<0.05 vs. the Normal group, ^b^*p*<0.05 vs. the HFpEF group.
