# Supplementary figures and images for: Dapagliflozin improves left ventricular remodeling and aorta sympathetic tone in a pig model of heart failure with preserved ejection fraction
Source: Cardiovasc Diabetol. 2019 Aug 20;18:107. doi: 10.1186/s12933-019-0914-1 (PMC6702744; doi:10.1186/s12933-019-0914-1)

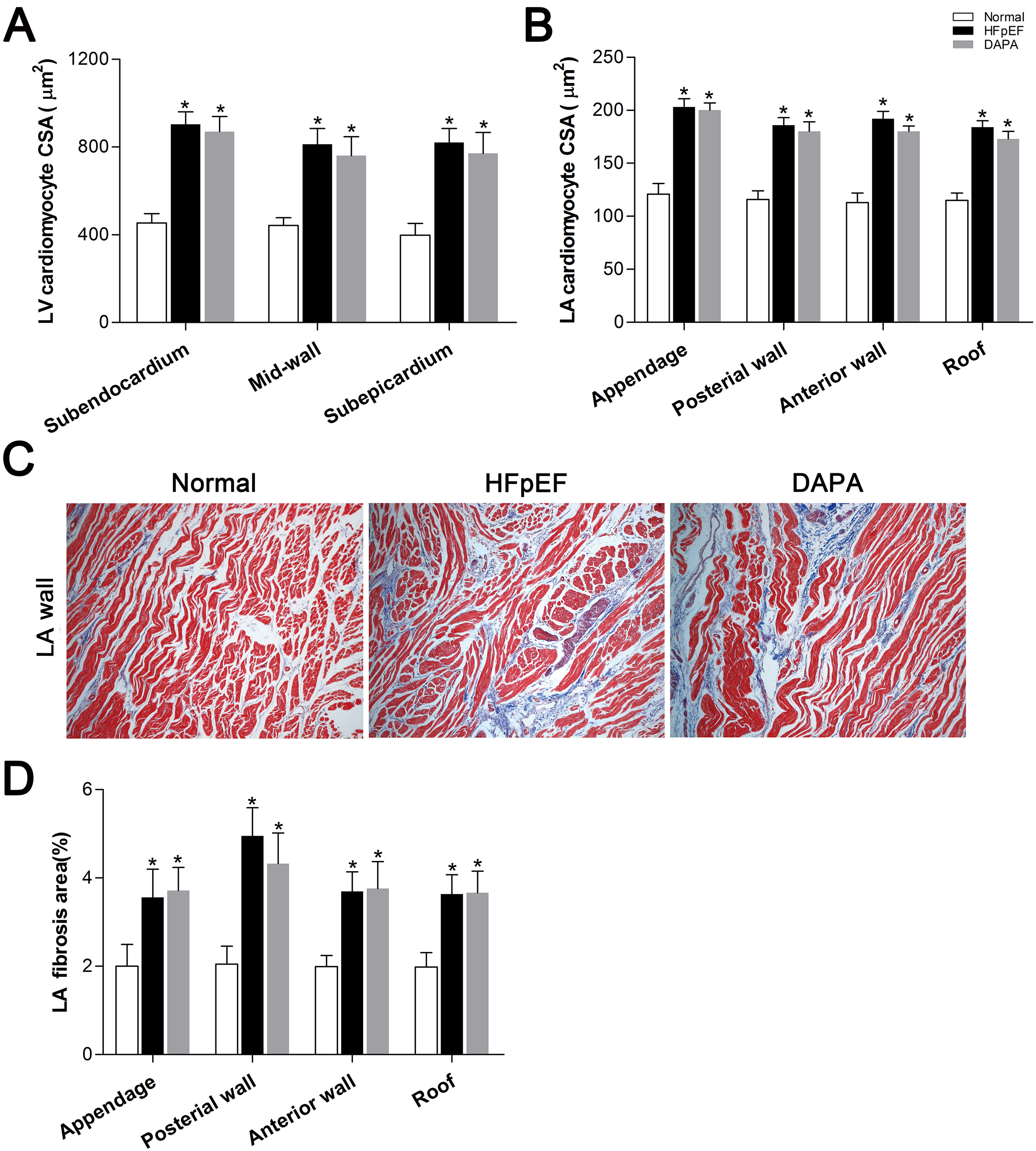

Supplement: Supplementary file 4 — Additional file 4: Fig. S1. Dapagliflozin had no significant effect on cardiomyocyte CSA or LA fibrosis in HFpEF pigs. The cardiomyocyte CSA of the LV and LA was calculated (A and B). Representative images of Masson’s trichrome staining of the LA posterior wall are shown (C). The area percentages of LA fibrosis were calculated (D). Values are expressed as the mean ± SD. n = 10 pigs per group. Statistical analyses were performed by one-way ANOVA followed by the Bonferroni post hoc test. *p < 0.05 vs. the Normal group. [file 12933_2019_914_MOESM4_ESM.tif]

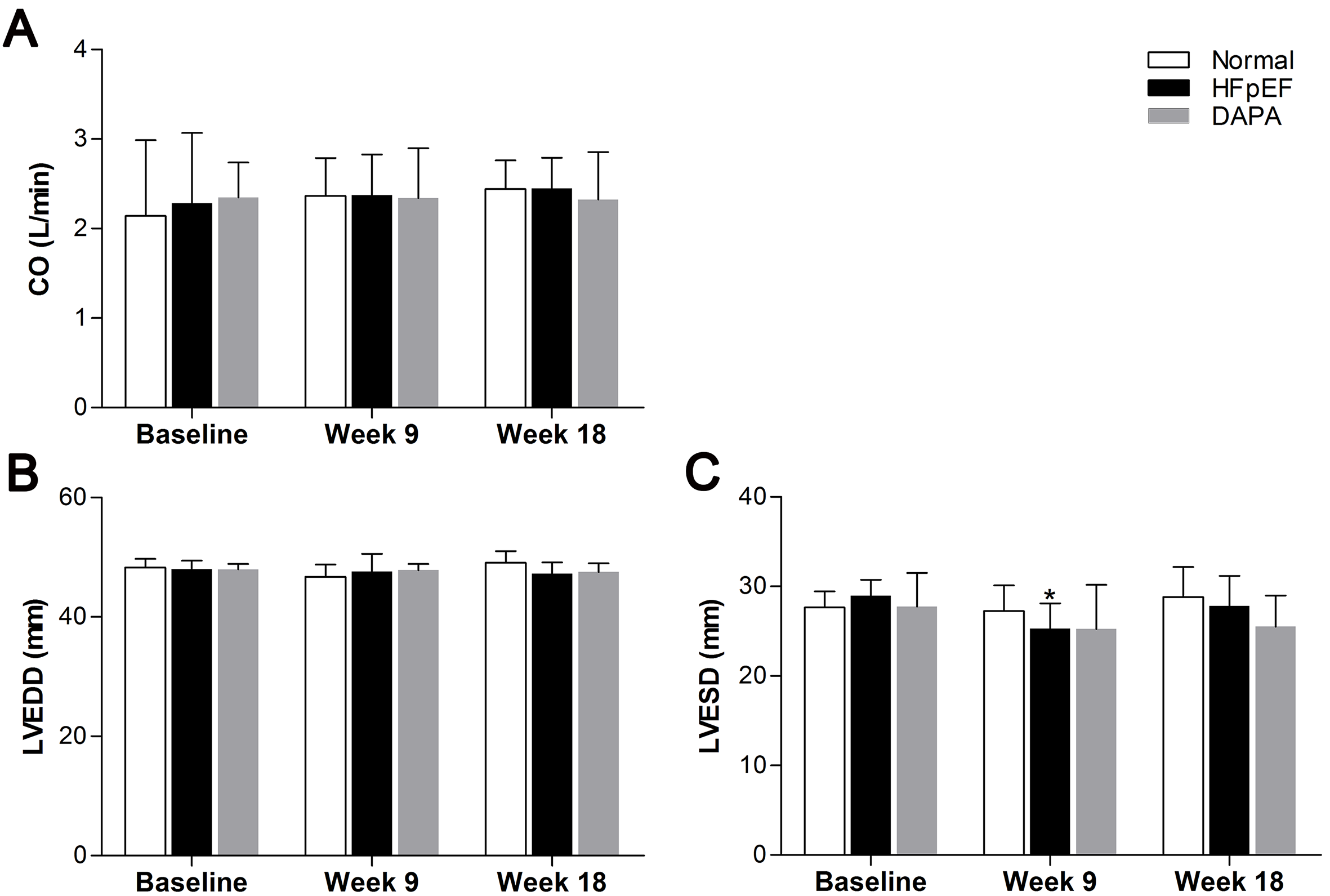

Supplement: Supplementary file 5 — Additional file 5: Fig. S2. Dapagliflozin had no significant effect on CO, LVEDD or LVESD in HFpEF pigs. CO, cardiac output; LVEDD, left ventricular end-diastolic dimension; LVESD, left ventricular end-systolic dimension. Values are expressed as the mean ± SD. n = 10 pigs per group. Statistical analyses were performed by one-way ANOVA followed by the Bonferroni post hoc test. *p < 0.05 vs. the Normal group. [file 12933_2019_914_MOESM5_ESM.tif]
